# Autophagy in plants: molecular mechanisms and roles in abiotic stress responses

**DOI:** 10.3389/fpls.2026.1861141

**Published:** 2026-06-18

**Authors:** Qiuyu Lv, Anfel Soltani, Mingguang Lei, Guojie Ma

**Affiliations:** College of Life and Environmental Sciences, Hangzhou Normal University, Hangzhou, China

**Keywords:** ATG genes, autophagy, organellar autophagy, selective autophagy, SnRK1, TOR signaling

## Abstract

Autophagy is an evolutionarily conserved catabolic pathway that maintains cellular homeostasis by degrading and recycling cytoplasmic components in the vacuole or lysosome. In plants, this process is indispensable for nutrient remobilization and stress acclimation, clearing dysfunctional organelles, protein aggregates, and other cytoplasmic material. This review covers current knowledge of plant autophagy, beginning with the core ATG (autophagy-related) machinery and the mechanisms of selective cargo recognition mediated by receptors such as NBR1 and the ATG8 protein family, then extending to organelle-specific degradation pathways including chlorophagy, mitophagy, and ER (endoplasmic reticulum)-phagy. We examine how autophagy supports plant survival under adverse conditions with attention to the regulatory networks governing autophagic activity, including the antagonistic TOR (Target of Rapamycin) and SnRK1 (Sucrose Non-Fermenting Related Kinase 1) kinase pathways that act as central nutrient and energy sensors. Collectively, these findings establish autophagy not merely as a cellular housekeeping mechanism, but as a central regulatory hub that coordinates plant growth with environmental adaptation, with important implications for engineering stress-resilient crops.

## 1 Introduction

As sessile organisms, plants must constantly adapt to fluctuating environmental conditions while sustaining growth and development. Among the key adaptive mechanisms, autophagy is a fundamental process that integrates cellular homeostasis with environmental stress acclimation (Marshall and Vierstra, 2018; Liu and Bassham, 2012). Literally meaning “self-eating”, autophagy is an evolutionarily conserved pathway involving the formation of double-membrane vesicles called autophagosomes, which engulf cytoplasmic components and deliver them to the vacuole for enzymatic degradation and nutrient recovery (Mizushima and Komatsu, 2011).

Three principal forms of autophagy are recognized in eukaryotes: macroautophagy, microautophagy, and chaperone-mediated autophagy (Galluzzi et al., 2017). Macroautophagy (hereafter referred to as autophagy) is the most extensively studied form in plants. This process involves coordinated membrane dynamics in which autophagosomes form, engulf cargo, and fuse with the vacuolar membrane to release their contents for degradation by acidic hydrolases (Feng et al., 2014; Zhuang et al., 2024). Under normal conditions, autophagy operates at a basal level for cellular maintenance, but it is strongly induced by nutrient deprivation, oxidative damage, temperature extremes, and pathogen attack, enabling efficient recycling of damaged components to sustain plant viability (Liu et al., 2009; Masclaux-Daubresse et al., 2017).

Plant autophagy research has moved well beyond basic mechanistic studies, with recent work clarifying how selective processes operate and why they matter physiologically. Li et al. (2024) showed that stress granules serve as transient reservoirs for autophagy proteins, enabling rapid autophagic recovery following heat stress. That work, alongside the characterization of organelle-specific degradation pathways, has fundamentally refined our understanding of selective cellular recycling in plants (Nakamura and Izumi, 2019; Stephani and Dagdas, 2020). The identification of selective autophagy receptors—NBR1 (Neighbor of BRCA1 gene 1) in particular—and their interactions with ATG8 family proteins has provided molecular insight into how cargo specificity is achieved (Ji et al., 2020; Lin et al., 2021).

This review consolidates current knowledge of plant autophagy and highlights recent discoveries that have expanded our mechanistic understanding. We address the core molecular machinery, abiotic stress responses, and regulatory networks, with emphasis on selective and organelle-specific autophagy pathways.

## 2 Autophagy machinery and selective cargo recognition

Autophagy was first observed in plants in the late 1960s by electron microscopy (Matile and Moor, 1968), yet its functional significance and mechanistic basis remained poorly understood for decades. The genetic identification of *ATG* (autophagy-related) genes in *Saccharomyces cerevisiae* (Tsukada and Ohsumi, 1993) provided the conceptual framework needed to dissect the pathway. At its core, autophagy is executed by a set of conserved ATG proteins and proceeds through sequential steps: induction, phagophore nucleation, phagophore elongation, autophagosome maturation, vacuolar fusion, and cargo degradation (Galluzzi et al., 2017).

### 2.1 Core autophagy machinery

**Figure 1:**
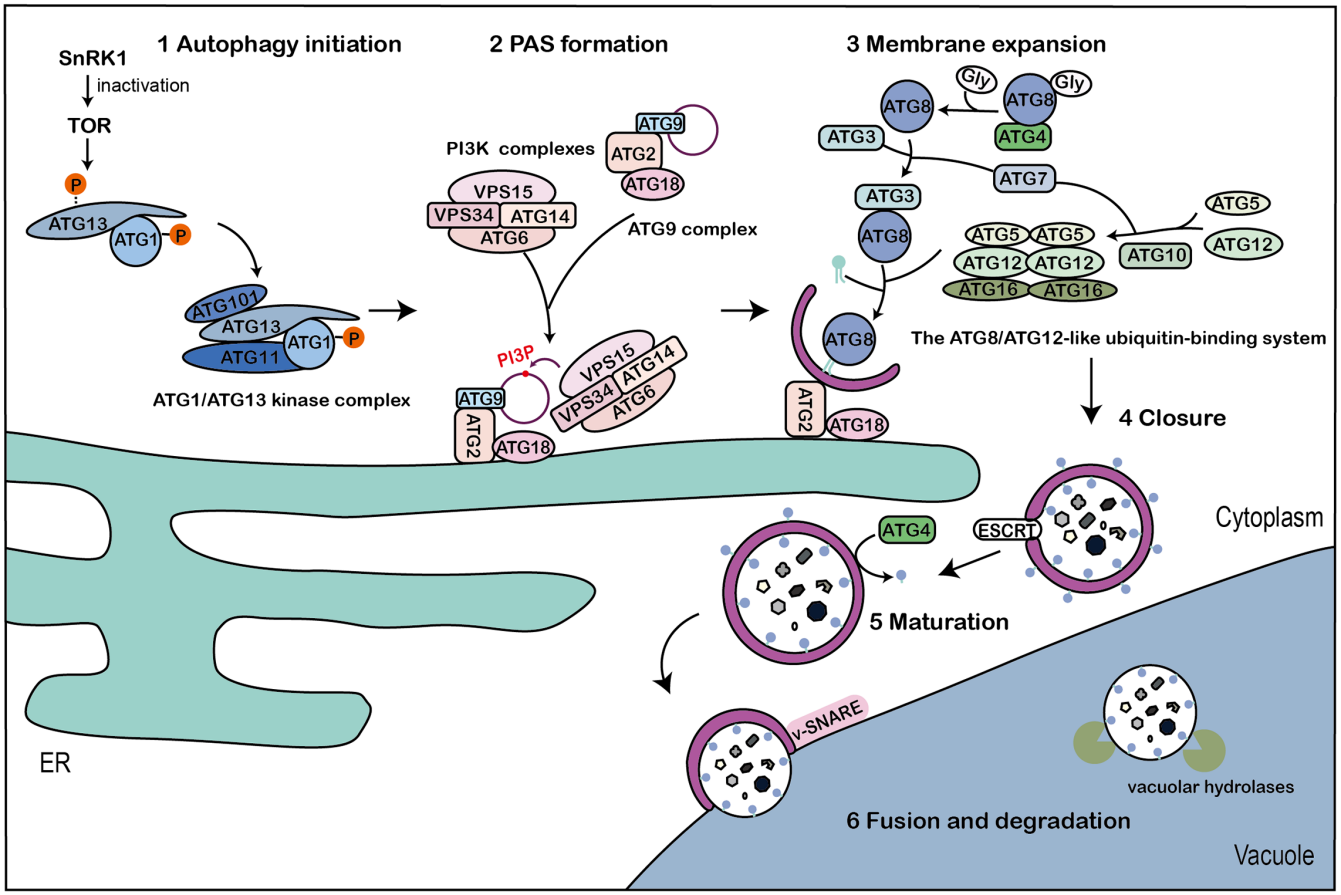
Key steps of plant autophagy. Autophagy initiation: Under stress conditions, the ATG1 kinase complex is activated, initiating autophagy. PAS formation: The PI3K complex generates PI3P at the phagophore assembly site (PAS), recruiting effector proteins. The phagophore scaffold is constructed using the endoplasmic reticulum membrane, ATG9 vesicles, and newly synthesized lipids. Membrane expansion: The ATG2-ATG9-ATG18 complex mediates lipid transport, with ATG9 providing flippase activity. ATG8 stabilizes the phagophore and recruits cargo. Closure: ESCRT components facilitate the fusion of the phagophore edges, resulting in a closed double-membrane autophagosome. Maturation: Most ATG proteins dissociate, while the inner membrane-associated ATG8-PE remains bound to the cargo. Fusion and degradation: The autophagosome fuses with the vacuole, delivering the cytoplasmic cargo into the vacuole for degradation and recycling.

The molecular machinery of plant autophagy is broadly conserved with that of other eukaryotes, though it incorporates several plant-specific features (Zhuang et al., 2024; Yagyu and Yoshimoto, 2024). The core ATG proteins are organized into four functionally distinct complexes, as illustrated in **Figure 1**.

#### 2.1.1 The ATG1/ATG13 kinase complex and upstream regulation

Autophagy initiation is governed by the ATG1/ATG13 kinase complex, which integrates cellular nutrient and energy signals. In plants, the SnRK1 complex (Sucrose Non-Fermenting Related Kinase 1) acts as a central metabolic sensor activated under energy or nutrient deficiency (Baena-González et al., 2007; Crozet et al., 2014). SnRK1 promotes autophagy primarily by suppressing TOR kinase (Target of Rapamycin) activity (Soto-Burgos and Bassham, 2017). Under nutrient-replete conditions, TOR may phosphorylate ATG13, preventing its association with ATG1. Upon nutrient deprivation, SnRK1-mediated inhibition of TOR is thought to lead to ATG13 dephosphorylation, enabling ATG13–ATG1 complex formation and ATG1 autophosphorylation (Son et al., 2018). The activated ATG1–ATG13 complex then recruits ATG11 and ATG101 to initiate downstream autophagy events (Soto-Burgos and Bassham, 2017; Wang and Hou, 2022; Suttangkakul et al., 2011; Li and Vierstra, 2014). An additional layer of control comes from the E3 ubiquitin ligases SINAT1 and SINAT2, which act together with TRAF1a and TRAF1b to target ATG6 for ubiquitin-mediated degradation, thereby fine-tuning autophagic flux (Qi et al., 2017, Qi et al., 2020). Additionally, the ATG1/ATG13 complex itself is subject to autophagic turnover, providing a negative feedback loop that limits autophagy induction (Suttangkakul et al., 2011).

#### 2.1.2 The PI3K complex and phagophore nucleation

Once the ATG1 complex is activated, phagophore nucleation proceeds through the coordinated action of the class III phosphatidylinositol 3-kinase (PI3K) complex and the ATG9 transmembrane protein (Kotani et al., 2018; Zhuang et al., 2017; Noda et al., 2000). The PI3K complex phosphorylates phosphatidylinositol to generate phosphatidylinositol-3-phosphate (PI3P) on the nascent phagophore membrane (Liu et al., 2020). PI3P serves as a recruitment signal for the ATG18–ATG2 complex: an amphipathic helix in the C-terminal region of ATG2 binds directly to membranes, while ATG18 binds PI3P to target the complex to the phagophore assembly site, together facilitating membrane tethering and expansion (Kotani et al., 2018). The PI3K complex exists in two distinct forms, both sharing the catalytic subunit VPS34 together with VPS15 and ATG6 (also known as VPS30 or Beclin1). In *Arabidopsis*, the VPS38 subunit present in one PI3K complex variant is dispensable for autophagy (Lee et al., 2018). Genetic dissection has revealed a temporal hierarchy: ATG11, ATG9, and the PI3K complex act at early stages of phagophore assembly, while ATG2 functions at a later step during autophagosome formation (Kang et al., 2018).

#### 2.1.3 The ATG8/ATG12 conjugation systems

Phagophore elongation and closure require two ubiquitin-like conjugation systems: the ATG8 and ATG12 systems. In the ATG8 lipidation cascade, the cysteine protease ATG4 cleaves the C-terminus of ATG8 to expose a conserved glycine residue; the E1-like enzyme ATG7 then activates ATG8 and transfers it to the E2-like enzyme ATG3, which conjugates ATG8 to the membrane lipid phosphatidylethanolamine (PE), anchoring ATG8 to the phagophore membrane (Fujioka et al., 2008; Ichimura et al., 2000). The ATG12 conjugation system runs in parallel: ATG7 activates ATG12, which is transferred to ATG10 and covalently attached to ATG5 (Fujioka et al., 2008). Two ATG12–ATG5 conjugates then associate with an ATG16 dimer to form a hexameric E3-like complex that promotes ATG8–PE conjugation (Chung et al., 2010; Hanada et al., 2007).

Recent studies have revealed functional specialization among ATG8 isoforms. Analysis of an *Arabidopsis* nonamer mutant lacking all nine *ATG8* genes showed that, although ATG8 proteins are largely functionally redundant, individual isoforms possess distinct capacities: ATG8A rescues growth under both carbon and nitrogen limitation, whereas ATG8H is effective only under carbon starvation (Del Chiaro et al., 2025). This isoform-specific divergence suggests that ATG8 family expansion in vascular plants has been accompanied by functional diversification, allowing more precisely calibrated stress responses. In a separate line of work, ATG8 delipidation—long considered essential for autophagosome biogenesis—turned out to be dispensable for autophagosome maturation and stress survival in *Arabidopsis*, yet remains critical in the green alga *Chlamydomonas reinhardtii*, pointing to an evolutionary divergence in the mechanistic requirements for autophagy between unicellular and multicellular plants (Zou et al., 2025).

A further regulatory mechanism involves an N-degron pathway that mediates ATG8 isoform switching during recovery from heat stress. The E3 ubiquitin ligase UBR7 selectively targets specific ATG8 isoforms for proteasomal degradation, enabling the cell to replace them with alternative isoforms and thereby calibrate autophagic activity to the appropriate level for thermotolerance acquisition (Kim et al., 2025; Kim and Park, 2025).

#### 2.1.4 Autophagosome maturation and cargo degradation

Once autophagosomes form, their delivery to the vacuole depends on specialized membrane trafficking machinery. The ESCRT (endosomal sorting complex required for transport) pathway, which canonically mediates ubiquitinated cargo sorting into multivesicular bodies for vacuolar delivery, also contributes to autophagic degradation in plants (Gao et al., 2015, Gao et al., 2017). The plant-specific ESCRT component FREE1 (FYVE domain protein required for endosomal sorting 1) operates in both vacuolar protein trafficking and autophagic degradation and interacts directly with SH3P2 to regulate the timing of autophagosome–vacuole fusion (Gao et al., 2015; Kolb et al., 2015; Zheng et al., 2026). Terminal fusion of autophagosomes with the vacuolar membrane is mediated by v-SNARE proteins, releasing autophagic bodies into the vacuolar lumen for degradation by resident hydrolases (Yang and Bassham, 2015; Jung et al., 2026). Among these hydrolases, vacuolar processing enzyme-γ (VPEγ) is upregulated during senescence and programmed cell death, where it initiates a proteolytic cascade that activates additional vacuolar enzymes (Rojo et al., 2003).

A recently characterized pathway involves VPS41-associated phagic vacuoles (VAPVs). Upon autophagy induction, the HOPS (homotypic fusion and protein sorting) tethering complex subunit VPS41 transitions from a dispersed condensate state into discrete puncta, which subsequently reorganize into ring-like VAPV structures that facilitate autophagosome–vacuole fusion (Jiang et al., 2024). Disrupting this pathway impairs both autophagosome biogenesis and vacuolar delivery, underscoring its importance for autophagic flux under stress conditions.

Beyond its role in vacuolar trafficking, FREE1 promotes phagophore closure under nutrient-limiting conditions by bridging the ATG conjugation system with the ESCRT machinery (Zeng et al., 2023). Separately, ATG18a has been shown to undergo liquid-liquid phase separation, forming dynamic droplets on the ER membrane that progressively mature into stable ring-like structures. This phase separation-driven ER remodeling creates membrane platforms that support autophagosome assembly (Shao et al., 2026).

### 2.2 Selective autophagy and cargo recognition

A major conceptual advance in plant autophagy research is the recognition that autophagic degradation is predominantly selective rather than indiscriminate (Marshall and Vierstra, 2018; Luo et al., 2021). Selective autophagy depends on receptor proteins that recognize specific cargo—either through ubiquitin-binding domains or via ubiquitin-independent signals—and deliver it to autophagosomes by interacting with ATG8 proteins through conserved ATG8-interacting motifs (AIM/LIR) (Lamark and Johansen, 2021; Wu et al., 2025b). The functional diversification of ATG8 isoforms in plants contributes to the specificity of these interactions (Del Chiaro et al., 2025).

NBR1 is the best-characterized selective autophagy receptor in plants, required for the selective clearance of protein aggregates and damaged organelles under stress conditions (Zhou et al., 2013, Zhou et al., 2014). Unlike animals, which possess both NBR1 and p62/SQSTM1 as distinct receptors, plants rely on a single NBR1 protein to perform both functions. This protein comprises an N-terminal PB1 oligomerization domain, a central coiled-coil region, an ubiquitin-associated (UBA) domain, and a C-terminal ATG8-interacting motif (AIM), enabling it to bridge ubiquitinated cargo with the autophagic machinery.

The documented cases of NBR1-mediated selective autophagy in plants reveal a remarkably versatile receptor that operates across diverse biological contexts (**Table 1**; Zhang and Chen, 2020; Luo et al., 2021). In the context of proteotoxic stress, NBR1 targets ubiquitinated protein aggregates for vacuolar degradation, acting additively with the E3 ubiquitin ligase CHIP to maintain proteostasis (Zhou et al., 2013, Zhou et al., 2014). During viral infection, NBR1 mediates antiviral autophagy by targeting coat proteins of Tobacco mosaic virus (TMV) and Turnip mosaic virus (TuMV), contributing to plant immunity (Hafrén et al., 2017). The ability of NBR1 to undergo liquid-liquid phase separation (LLPS) extends its substrate range beyond ubiquitinated cargo to include condensate-forming proteins, particularly under heat stress conditions where NBR1-driven condensates are targeted for autophagic disposal (Yan et al., 2024). Under heat stress, ERC1 (ELKS/Rab6-interacting/CAST family member 1) provides another route to autophagosome biogenesis by forming biomolecular condensates that recruit ATG8 and NBR1 to drive autophagosome assembly at elevated temperatures (Chung et al., 2025). In developmental contexts, NBR1 participates in hormone signaling: in maize, ZmNBR1 promotes autophagic degradation of the brassinosteroid receptor ZmBRI1a to modulate BR signaling under drought stress (Xiang et al., 2024), while in Arabidopsis, NBR1 targets the exocyst subunit AtExo70E2 for selective degradation (Ji et al., 2020). NBR1 also functions in heavy metal detoxification through its interaction with HIPP33, which bridges cadmium-associated cargo to the autophagic machinery (Wu et al., 2025a). Furthermore, NBR1 participates in the N-degron pathway, recognizing substrates tagged by the UBR7 E3 ligase for selective autophagic clearance (Marshall et al., 2015). In poplar, overexpression of PtNBR1 enhances salt stress tolerance by promoting selective autophagy of stress-damaged proteins (Su et al., 2021). The breadth of NBR1 cargo—spanning proteotoxic aggregates, viral particles, hormone receptors, exocyst components, heavy metal-associated proteins, and N-degron substrates—establishes NBR1 as a central hub receptor in plant selective autophagy.

**Table 1:** Documented cases of NBR1-mediated selective autophagy in plants.

Cargo	Organism	Context	Mechanism	Reference
Ubiquitinated protein aggregates	Arabidopsis	General stress/proteotoxic	NBR1 UBA domain binds poly-Ub; PB1 domain oligomerizes; delivers to autophagosome via ATG8 LIR	Zhou et al., 2013; Marshall and Vierstra, 2018
Viral coat proteins (TMV, TuMV)	Arabidopsis	Viral infection	NBR1 targets viral particles for autophagic clearance; antiviral defense	Hafrén et al., 2017
LLPS condensates/stress granule components	Arabidopsis	Heat/proteotoxic stress	AtNBR1 drives liquid-liquid phase separation; condensates delivered to vacuole	Yan et al., 2024
ZmBRI1a (brassinosteroid receptor)	Maize	Developmental/stress	ZmNBR1 promotes autophagic degradation of ZmBRI1a; regulates BR signaling	Xiang et al., 2024
Cadmium-associated proteins via HIPP33	Arabidopsis	Heavy metal stress	HIPP33 bridges cadmium-associated cargo to NBR1; vacuolar sequestration	Wu et al., 2025a
AtExo70E2 (exocyst subunit)	Arabidopsis	Developmental	AtNBR1 is selective receptor for AtExo70E2	Ji et al., 2020
UBR7-dependent N-degron substrates	Arabidopsis	N-degron pathway	UBR7 E3 ligase generates N-degron signals recognized by NBR1	Marshall et al., 2015
Insoluble ubiquitinated aggregates under proteasome inhibition	Arabidopsis	Proteotoxic stress	NBR1 + CHIP E3 ligase act additively	Zhou et al., 2014
Poplar NBR1 targets under salt stress	Poplar	Salt stress	PtNBR1 overexpression enhances salt tolerance via selective autophagy	Su et al., 2021

### 2.3 Organellar autophagy pathways

#### 2.3.1 Chlorophagy

Chlorophagy—the selective autophagic degradation of chloroplasts or chloroplast-derived material—is a plant-specific pathway active during leaf senescence, nutrient limitation, and environmental stress (Wada et al., 2009; Ishida and Yoshimoto, 2008). Two mechanistically distinct routes have been described: an ATG-dependent pathway that employs the core autophagy machinery to engulf chloroplasts within autophagosomes, and an ATG-independent pathway that delivers chloroplast components directly to the vacuole via specialized vesicles (Otegui, 2018). Entire photodamaged chloroplasts can be transported to the central vacuole through the ATG-dependent route (Izumi et al., 2017), while smaller chloroplast-derived structures known as Rubisco-containing bodies (RCBs) are delivered to the vacuole via ATG-dependent processes during carbon limitation and senescence (Ishida et al., 2008).

Recent studies have substantially advanced our mechanistic understanding of chlorophagy. Live-cell imaging has demonstrated that autophagosome formation and chloroplast segmentation occur synchronously during piecemeal chloroplast degradation (Izumi et al., 2024). A notable biotechnological advance was the engineering of a synthetic chlorophagy receptor, LIR-SNT-BFP, which fuses an ATG8-interacting region with a chloroplast-targeting signal to promote chloroplast microautophagy. Expression of this synthetic receptor enhanced plant fitness and attenuated herbicide-induced chlorosis, though excessive receptor activity impaired growth and reduced chlorophyll content, highlighting the importance of balanced autophagic flux (Liu et al., 2025a).

An interaction between APE1 (ACCLIMATION OF PHOTOSYNTHESIS TO ENVIRONMENT1) and ATI1 (ATG8-INTERACTING PROTEIN1) within chloroplast stromules has been identified, suggesting a role for stromule-mediated signaling in selective chloroplast degradation during stress acclimation (Ge et al., 2025). Autophagy also plays a dual role in chromoplast biogenesis and degradation, processes that are essential for fruit pigmentation and ripening in tomato (Guo et al., 2025).

#### 2.3.2 Mitophagy

Mitophagy—the selective autophagic removal of mitochondria—serves as a key quality control mechanism that eliminates dysfunctional organelles while preserving the functional mitochondrial population (Ma et al., 2021; Li et al., 2022; Kacprzak and Van Aken, 2023). In plants, the pathway is induced by nutrient starvation, oxidative stress, and developmental senescence, and follows a conserved logic: damaged mitochondria are recognized by selective receptors, sequestered within autophagosomes, and delivered to the vacuole for degradation (Schmitt and Van Aken, 2023). The scaffold protein ATG11 is required for both general autophagy and senescence-associated mitophagy in Arabidopsis, linking the core autophagic machinery to organelle-specific degradation (Li et al., 2014).

Two proteins have emerged as key regulators of plant mitophagy: FRIENDLY and the TraB family. FRIENDLY is a cytosolic member of the conserved CLUSTERED MITOCHONDRIA (CLU) superfamily required for dark-induced mitophagy and the controlled progression of senescence (El Zawily et al., 2014; Kacprzak and Van Aken, 2023). Upon mitochondrial damage, FRIENDLY promotes the selective autophagic clearance of depolarized mitochondria, and friendly mutants show impaired mitophagy flux under stress conditions (Ma et al., 2021). FRIENDLY activity is subject to post-translational regulation through lysine acetylation at residues K1022 and K1029, which modulates intermitochondrial association and, consequently, mitophagy efficiency (El Zawily et al., 2014).

The TraB family proteins TRB1 and TRB2 function as dual-role regulators that couple ER-mitochondrial contact sites (EMCSs) to mitophagy (Li et al., 2022). As mitochondrial outer membrane (OMM) proteins, TRB1 and TRB2 contain two ATG8-interacting motifs (AIMs) and a conserved TraB-homology domain. Through interaction with the ER-localized VAP27–1 protein, they physically tether mitochondria to the ER membrane, forming EMCSs whose integrity is a prerequisite for efficient stress-induced mitophagy. In parallel, TRB1 engages ATG8 via its AIM motifs and recruits ATG8-labelled autophagosome membranes to depolarized mitochondria following DNP-induced damage. TRB1 overexpression promotes autophagic flux, while TRB1 degradation itself is autophagy-dependent, establishing TRB1 as both a mitophagy receptor and an autophagic substrate (Li et al., 2022). Together, FRIENDLY and the TraB proteins reveal that mitochondrial structural integrity and ER-mitochondrial communication are prerequisites for efficient mitophagy in plants.

#### 2.3.3 ER-phagy

ER-phagy—the selective autophagic degradation of endoplasmic reticulum (ER) subdomains—serves as an essential quality control mechanism in plants (Liu et al., 2012). Specialized ER-phagy receptors, including ATI1 (ATG8-INTERACTING PROTEIN 1) and ATI2, recognize ER-resident substrates and recruit them to autophagosomes through direct interaction with ATG8 proteins (Wu et al., 2021; Hu et al., 2020). This pathway is particularly critical during ER stress, when accumulation of misfolded proteins triggers selective ER degradation to restore proteostasis (Liu et al., 2012).

Studies of the dynamic association between autophagosomes and the ER have shed light on how ER-phagy is regulated. RABC1, a plant-specific member of the RABC/RAB18 GTPase family, regulates the sequential binding and detachment of autophagosomes from the ER membrane during autophagy induction, a process relevant to ER-phagy regulation (Shao et al., 2024; Shao et al., 2026). This RAB GTPase-mediated tethering is required for efficient autophagic flux under nutrient-limiting conditions. The importance of ER homeostasis has also been highlighted in the context of plant immunity: pathogens deploy effector proteins to subvert host ER functions, while plants counteract this through ER stress responses and ER-phagy (Liu et al., 2025b).

### 2.4 Stress granules and autophagy protein dynamics

Stress granules function as transient reservoirs for autophagy proteins during cellular stress (Li et al., 2024). Upon heat stress, key components of the ATG1/ATG13 kinase complex are sequestered within stress granules. This sequestration prevents excessive autophagic activity during the acute stress phase while maintaining the autophagy machinery in a primed state for rapid reactivation during recovery—a buffering strategy that calibrates autophagic output to the severity and duration of stress.

Autophagy components are also recruited to stress granules via liquid-liquid phase separation during heat stress (Feng et al., 2025). This dynamic partitioning balances immediate stress survival with post-stress recovery capacity. More broadly, these findings position biomolecular condensates as a regulatory layer in the control of plant autophagy.

## 3 Autophagy in abiotic stress responses

Plants are constantly exposed to fluctuating environmental conditions and must adapt morphologically, physiologically, and metabolically to maintain productivity. Abiotic stresses impose severe constraints on growth and yield, with major consequences for agricultural production (Signorelli et al., 2019; Zhu, 2016). Autophagy promotes stress tolerance by clearing damaged macromolecules and organelles, thereby maintaining cellular homeostasis. Plants deficient in autophagy exhibit markedly increased sensitivity to abiotic stress (Marshall and Vierstra, 2018).

### 3.1 Nutrient stress and starvation responses

Nutrient deprivation is among the most potent inducers of autophagy in plants (Xiong et al., 2005; Doelling et al., 2002). Under nutrient-limiting conditions, autophagy is strongly induced to mobilize intracellular reserves through the degradation of stored macromolecules and organelles, including 26S proteasomes and entire chloroplasts (Wada et al., 2009; Marshall et al., 2015). The magnitude of this response is illustrated by the observation that sucrose-starved suspension-cultured cells degrade 30–50% of their total protein within 48 hours (Moriyasu and Ohsumi, 1996).

Under carbon starvation, *Arabidopsis atg5* and *atg7* mutants display impaired growth, reduced free amino acid levels, elevated respiration rates, and compromised protein synthesis (Avin-Wittenberg et al., 2015). Under nitrogen starvation, *atg* mutants accumulate ammonium, amino acids, and proteins in leaves (Guiboileau et al., 2013), while ATG8-mediated autophagy delivers 26S proteasomes to the vacuole for degradation (Marshall et al., 2015). Conversely, overexpression of *ATG8* family genes in *Arabidopsis*, soybean, foxtail millet, and rice promotes growth, enhances tolerance to nitrogen starvation and drought, and improves yield (Chen et al., 2019; Li et al., 2015, Li et al., 2016; Xia et al., 2012; Yu et al., 2019).

Beyond carbon and nitrogen, autophagy contributes to the homeostasis of other nutrients. It regulates the balance between zinc and iron uptake (Shinozaki and Yoshimoto, 2021) and enhances zinc bioavailability under zinc-deficient conditions (Shinozaki et al., 2020). Autophagy also facilitates sulfur remobilization: *atg5* mutants are impaired in the transfer of sulfur from rosette leaves to seeds (Lornac et al., 2020).

Recent work has implicated VPS9a in autophagy-dependent nutrient recycling. Loss of VPS9a function confers hypersensitivity to both carbon and nitrogen starvation and disrupts the glutamine synthetase/glutamate synthase (GS/GOGAT) cycle, revealing a functional link between endosomal sorting and nitrogen metabolism during autophagy-mediated recycling (Yu et al., 2025).

### 3.2 Drought stress

Drought stress severely constrains plant growth and threatens crop yield and food security (Seleiman et al., 2021; Lamaoui et al., 2018). The connection between autophagy and drought tolerance was first established in *Arabidopsis*, where osmotic stress upregulates *ATG18a* expression and stimulates autophagosome formation (Liu et al., 2009). Knockdown of *ATG5*, *ATG7*, or *ATG18a* attenuates autophagy and reduces drought tolerance (Liu et al., 2009; Zhou et al., 2013).

Drought-responsive induction of *ATG* genes has been documented across diverse crop species, including *ATG8a* in foxtail millet (Li et al., 2016), *ATG6* in barley (Zeng et al., 2017), and *ATG3* and *ATG18a* in apple (Wang et al., 2014; Wang et al., 2017). Overexpression of apple *MdATG18a* in tomato enhances autophagic activity and improves drought tolerance (Sun et al., 2018). In tomato, the heat-inducible transcription factor *HsfA1a* binds directly to the promoters of *ATG10* and *ATG18f* to activate autophagy during drought stress (Wang et al., 2015). In *Arabidopsis*, the plant-specific protein AtCOST1 (CONSTITUTIVELY STRESSED 1) suppresses drought responses by binding ATG8 and inhibiting its activation under normal conditions. When water is deficient, AtCOST1 is degraded, releasing ATG8 to induce autophagy (Bao et al., 2020).

A further regulatory connection was established with the identification of SRAS1.1, an E3 ubiquitin ligase that controls DSK2A stability, thereby linking the ubiquitin-proteasome system with autophagy induction during drought stress (Li et al., 2025). Overexpression of *StATG8a* in potato confers improved drought and salt tolerance, with transgenic plants exhibiting substantially greater stress tolerance than controls (Zhu et al., 2024). Similarly, overexpression of *ZmATG3* in maize improves tolerance to multiple abiotic stresses, including salt, mannitol, and nitrogen deficiency (Liu et al., 2024). In cotton, GhATG8f enhances salt tolerance by upregulating antioxidant enzyme activities (SOD, POD, and CAT) and promoting proline accumulation (Chen et al., 2024).

Melatonin has emerged as a regulator of autophagy under drought conditions. In drought-sensitive cotton, melatonin-mediated autophagy induction operates independently of abscisic acid (ABA) signaling, improving growth, chlorophyll content, photosynthetic efficiency, and sugar metabolism without altering ABA levels (Supriya et al., 2024).

### 3.3 Temperature stress

#### 3.3.1 Heat stress

Elevated temperatures impair plant growth by inducing abnormal protein folding and denaturation (Guihur et al., 2022). Plants respond by upregulating autophagy to clear toxic protein aggregates. Heat stress induces *ATG* gene expression and increases autophagosome abundance across multiple species (Zhou et al., 2013; Cheng et al., 2016; Rana et al., 2012). Silencing *ATG5* or *ATG7* in *Arabidopsis* and tomato markedly increases heat sensitivity (Zhou et al., 2013, Zhou et al., 2014; Cheng et al., 2016).

The discovery that stress granules serve as transient reservoirs for autophagy proteins during heat stress represents a key mechanistic advance (Li et al., 2024). Sequestration of ATG1 and ATG13 within stress granules prevents excessive autophagic activity during acute heat stress while preserving the capacity for rapid autophagic reactivation during recovery. The selective autophagy receptor NBR1 cooperates with ATG8 to target heat-damaged proteins for removal (Zhou et al., 2013), and *atg* and *nbr1* mutants are hypersensitive to heat and accumulate insoluble ubiquitinated protein aggregates (Zhou et al., 2013, Zhou et al., 2014). ATG8 also interacts with heat shock proteins (HSPs), and autophagy disruption leads to HSP accumulation, suggesting that autophagy contributes to their turnover (Sedaghatmehr et al., 2019). In apple, overexpression of *MdATG18a* improves basal thermotolerance by reducing chloroplast damage, improving photosynthetic performance, and increasing antioxidant capacity (Huo et al., 2020b). In tomato, overexpression of *SlATG8f* enhances autophagy and protects pollen viability during heat stress, a finding with direct implications for maintaining crop fertility under high-temperature conditions (Song et al., 2024).

#### 3.3.2 Cold stress

Low temperatures also induce autophagy, a response associated with membrane rigidification and elevated reactive oxygen species (ROS) production (Valitova et al., 2019). Cold stress activates autophagy through multiple converging mechanisms, including altered membrane fluidity, metabolic slowdown, and protein dysfunction. In tomato, strigolactones stabilize the transcription factor HY5 (ELONGATED HYPOCOTYL 5), which directly binds the *ATG18a* promoter to activate autophagy gene expression and promote autophagosome formation, facilitating clearance of cold-induced protein aggregates (Chi et al., 2020, Chi et al., 2025). This strigolactone-HY5 module also drives degradation of ubiquitinated proteins, extending its protective function beyond aggregate clearance. Within the brassinosteroid signaling pathway, BZR1 (BRASSINAZOLE-RESISTANT 1) induces autophagy by transcriptionally activating *NBR1* and *ATG* genes, contributing to cold tolerance (Chi et al., 2020).

### 3.4 Salt stress

Soil salinity is a major constraint on global agricultural productivity (Flowers, 2004; Munns and Tester, 2008). High salinity reduces photosynthesis, increases energy expenditure, and promotes ROS accumulation. Salt stress induces autophagy, which provides recycled building blocks and energy by degrading damaged proteins and organelles. In wheat, disruption of ATG2 or ATG7 impairs autophagy and reduces salt tolerance (Yue et al., 2021). Silencing the wheat metacaspase *TaMCA-Id* reduces salt tolerance in seedlings by promoting ROS accumulation, implicating this enzyme in the regulation of salt-induced autophagy and programmed cell death (Yue et al., 2022). In *Arabidopsis*, the PI3K complex enhances salt tolerance by facilitating internalization of the plasma membrane aquaporin PIP2;1 under salt stress, thereby reducing root water permeability (Ueda et al., 2016). Overexpression of *MdATG10* in apple roots increases autophagic activity and confers improved salt tolerance (Huo et al., 2020a).

### 3.5 Oxidative stress

ROS accumulation is a common consequence of multiple abiotic stresses, and autophagy is a central component of the cellular response to oxidative damage (Xiong et al., 2007; Cadena-Ramos et al., 2026). Autophagy both responds to and modulates ROS levels by selectively degrading ROS-generating organelles and recycling antioxidant components. The underlying mechanisms include oxidative modifications to autophagy regulatory proteins and activation of ROS-sensitive signaling cascades. By routing damaged chloroplasts and mitochondria to degradation, autophagy is essential for maintaining cellular redox homeostasis.

### 3.6 Circadian regulation of autophagy

Autophagic activity is not simply constitutive or stress-triggered—it follows a diurnal rhythm governed by the circadian clock (Supriya et al., 2025). Core clock components, including TOC1, PRRs, COR, and LUX, impose time-of-day restrictions on autophagy-related gene expression, generating oscillatory patterns of autophagic activity. This circadian gating likely synchronizes nutrient recycling with the plant’s daily metabolic cycles and offers potential strategies for optimizing crop performance.

## 4 Regulatory networks controlling autophagy

### 4.1 Transcriptional regulation

Autophagy gene expression is controlled by complex transcription factor networks that integrate developmental and environmental signals (Wang et al., 2026). Key regulators include members of the NAC, WRKY, and bZIP transcription factor families. Among these, the heat-inducible factor HsfA1a directly activates *ATG10* and *ATG18f* expression during drought stress (Wang et al., 2015). Recent work has shown that the phytochrome-interacting factors PIF4 and PIF5 directly bind the promoters of *ATG5*, *ATG12*, and *ATG8* genes to activate their transcription during leaf senescence, linking light signaling to autophagic activity in aging tissues (Lee et al., 2025).

A major advance is the identification of the SnRK1–JMJ15–CRF6 regulatory module, which integrates energy sensing with mitochondrial signaling to balance growth and oxidative stress responses (Zhao et al., 2025). In this module, SnRK1, acting as the central energy sensor, phosphorylates both the histone demethylase JMJ15—which removes histone H3K4me3 marks from target loci—and the transcription factor CRF6, revealing an epigenetic mechanism for nucleus-mitochondria communication during stress adaptation.

### 4.2 Post−translational regulation

Post-translational modifications provide a rapid mechanism for autophagy regulation (Petersen et al., 2024). TOR suppresses autophagy initiation under nutrient-replete conditions primarily through direct phosphorylation of ATG13, which inhibits ATG1 complex assembly and activity; whether TOR directly phosphorylates ATG1 itself in plants remains less clearly established than in yeast (Soto-Burgos and Bassham, 2017; Son et al., 2018). Additional regulatory modifications include ubiquitination, acetylation, and ATG8 deconjugation. The E3 ubiquitin ligases SINAT1 and SINAT2 control autophagic flux by directing ATG13 and ATG6 toward ubiquitin-mediated degradation (Qi et al., 2017, Qi et al., 2020). ATG8 is conjugated to phosphatidylethanolamine (PE), catalyzed by the ATG7–ATG3–ATG12/ATG5/ATG16 cascade. This lipidation is required for autophagosome biogenesis, while the cysteine protease ATG4 acts as a deconjugase that recycles ATG8 from the outer autophagosomal membrane after closure (Chung et al., 2010; Hanada et al., 2007; Fujioka et al., 2008).

Acetylation of autophagy proteins has emerged as a regulatory mechanism in plants, with recent work showing that it alters both the activity and protein-protein interactions of autophagy machinery components (Huang and Guo, 2024). Rab3GAP-like (Rab3GAPL) has also been identified as a negative regulator of autophagy. It binds ATG8 and inactivates the small GTPase Rab8a, which is required for autophagosome trafficking. This finding provides new mechanistic insight into the membrane transport processes that underlie both autophagy and plant immunity (Yuen et al., 2024).

### 4.3 Hormonal regulation

Plant hormones integrate developmental programs with environmental stress responses in the regulation of autophagy (Liao and Bassham, 2020). ABA is a major positive regulator of autophagy during both stress and senescence. Its signaling proceeds through PYR/PYL receptors, PP2C phosphatases, and SnRK2 kinases, which ultimately modulate TOR activity to promote autophagy induction (Chen et al., 2020; Cutler et al., 2010; Park et al., 2009; Wang et al., 2018). Brassinosteroids regulate autophagy through the transcription factor BZR1, which transcriptionally activates *NBR1* and *ATG* genes (Chi et al., 2020). Ethylene contributes to drought tolerance through mitochondrial alternative oxidase-dependent autophagy (Zhu et al., 2018). Auxin modulates autophagy primarily via TOR signaling: the GTPase ROP2 binds and activates TOR in response to auxin, thereby suppressing autophagy induction (Schepetilnikov et al., 2017).

### 4.4 Energy regulation

The functional link between mitochondrial activity and central energy signaling is further supported by evidence connecting cytochrome c levels to SnRK1 pathway activity. Plants with reduced cytochrome c exhibit altered SnRK1 signaling, establishing a direct molecular link between mitochondrial respiratory function and the primary energy-sensing pathway that modulates autophagy (Coronel et al., 2025). The interplay among hexokinase (HXK), SnRK1, and TOR constitutes a critical regulatory network that coordinates plant growth and stress responses (Li and Zhao, 2024; Eom et al., 2024). Together, these three sugar-sensing components form an integrated signaling web that couples sugar availability to autophagy control, providing multiple potential targets for improving crop stress tolerance and nutritional quality.

## 5 Crop improvement and biotechnological applications

Mechanistic insights into plant autophagy have opened promising avenues for crop improvement. Targeted manipulation of autophagy components—particularly overexpression of *ATG8* and *NBR1*—has consistently enhanced stress tolerance across diverse plant species, improving drought resilience, salt tolerance, and performance under nutrient-limiting conditions (Su et al., 2021; Zhang and Chen, 2020). For example, overexpression of *MdATG18a* in apple improves both drought tolerance and basal thermotolerance (Sun et al., 2018; Huo et al., 2020b), and overexpression of *MdATG10* confers enhanced salt tolerance (Huo et al., 2020a).

Autophagy is central to nutrient remobilization in plants. In rice, overexpression of *OsATG8c* promotes nitrogen recycling, increasing both yield and nitrogen use efficiency (Zhen et al., 2019). Engineering crops with precisely tuned autophagy-mediated nutrient recycling represents a sustainable strategy for improving agricultural productivity, particularly on nutrient-poor soils where reducing fertilizer inputs is a priority.

The contribution of autophagy to crop resilience under environmental stress has been comprehensively reviewed (Agbemafle et al., 2025). As climate change intensifies heat, drought, and salinity stress, autophagy-mediated clearance of damaged organelles becomes increasingly critical for maintaining cellular function. Emerging approaches include treatment with raffinose, a trisaccharide that induces autophagy and increases plant biomass and yield, suggesting that modulation of sugar signaling may be a practical strategy for improving crop performance under stress (Magen et al., 2025). Autophagy induction during plant grafting also promotes wound healing, with direct implications for improving horticultural practices (Kurotani et al., 2025).

A key caveat has emerged from recent work: both insufficient and excessive autophagy can cause premature leaf senescence, likely as a consequence of impaired nutrient homeostasis (James et al., 2025). This finding underscores that optimal crop performance requires precise calibration of autophagic activity rather than simple upregulation, and that future engineering strategies must account for the dose-dependent consequences of autophagy modulation.

## 6 Summary

Plant autophagy has emerged as a central cellular process that integrates growth, development, and stress adaptation through sophisticated, multi-layered regulatory networks. Major advances include the discovery of novel selective autophagy receptors, the mechanistic characterization of organelle-specific degradation pathways, and the identification of regulatory mechanisms such as stress granule-mediated sequestration of autophagy proteins. The functional specialization of ATG8 isoforms—exemplified by N-degron-mediated isoform switching for thermotolerance—highlights the functional diversification of the ATG8 family beyond its canonical scaffolding role (Kim and Park, 2025). The identification of HIPP33 as a cadmium detoxification receptor, the dual roles of NBR1 in phase separation and cargo recognition, and the characterization of TraB family proteins as dual-function EMCS tethering factors and mitophagy receptors have substantially broadened our understanding of substrate specificity and organelle quality control in autophagic degradation (Wu et al., 2025a; Yan et al., 2024; Li et al., 2022).

Current evidence establishes that autophagy enhances stress tolerance through two complementary mechanisms: maintenance of cellular homeostasis via degradation of damaged components, and provision of recycled nutrients and energy. The deep integration of autophagy with hormone signaling, transcriptional networks, and central metabolism underscores its pivotal role in plant biology. The TOR–SnRK1 axis remains the primary hub for nutrient and energy sensing that calibrates autophagic activity, with additional regulatory layers provided by transcriptional control, post-translational modifications including acetylation, and hormonal signals. Emerging regulatory modules such as SnRK1–JMJ15–CRF6 reveal the depth of connectivity between energy signaling, epigenetic regulation, and mitochondrial function (Zhao et al., 2025).
